# Role of the Btk-PLC*γ*2 Signaling Pathway in the Bone Destruction of Apical Periodontitis

**DOI:** 10.1155/2019/8767529

**Published:** 2019-07-25

**Authors:** Lina Wang, Hong Zhang, Ming Dong, Meina Zuo, Shuo Liu, Ying Lu, Weidong Niu

**Affiliations:** Department of Endodontics and Periodontics, College of Stomatology, Dalian Medical University, Dalian, Liaoning Province, China

## Abstract

Chronic apical periodontitis is characterized by alveolar bone absorption in the apical region and is the result of the participation of various inflammatory mediators. Studies have shown that the Bruton tyrosine kinase- (Btk-) phospholipase C*γ*2 (PLC*γ*2) signaling pathway plays an important role in bone absorption, but it is unknown whether it plays a role in apical periodontitis bone destruction. Therefore, this study verified the role of Btk and PLC*γ*2 in bone resorption of apical periodontitis by *in vivo* and *in vitro* experiments. In the *in vivo* experiment, a mice model of apical periodontitis was established; apical bone resorption was confirmed by the numbers of osteoclasts and HE staining. Btk, PLC*γ*2, and nuclear factor of activated T-cells 1 (NFATc-1) were detected by immunohistochemical staining. In the *in vitro* experiment, lipopolysaccharides (LPS) were used to stimulate osteoclast precursor cell RAW264.7 to establish an inflammatory microenvironment and detect osteoclast differentiation. By silencing Btk, the expression of Btk, PLC*γ*2, and NFATc-1 was detected by real-time qPCR and Western blot, and osteoclastogenesis was detected by enzyme histochemical staining to further confirm the role of Btk in bone resorption. It was found that the expression of Btk, PLC*γ*2, and NFATc-1 changed significantly with the progression of inflammation and bone destruction, indicating that Btk and PLC*γ*2 may be involved in the progression of inflammation in apical periodontitis and bone absorption. *In vitro* experiments confirmed that the differentiation of osteoclasts and the expression of PLC*γ*2 and NFATc-1 were significantly inhibited after silencing Btk expression, but osteoclast precursor cells could be differentiated due to the proinflammatory factor lipopolysaccharide. This study demonstrates that Btk and PLC*γ*2 are key factors involved in the apical inflammatory response and bone destruction.

## 1. Introduction

Chronic apical periodontitis is caused by polymicrobial infection, such as bacterial, viral, and fungal [1], and alveolar bone resorption in the apical region is the main characteristic, which is the result of the participation of multiple inflammatory mediators [2]. Bruton tyrosine kinase (Btk) is a member of the nonreceptor Tec tyrosine kinase family, and these kinases are predominantly expressed in lymphocytes and myeloid cells [3]. When the nonreceptor tyrosine kinase binds to the receptor, it activates the downstream substrate to cause tyrosine phosphorylation and the signaling pathway [4]. Btk is involved in a variety of signaling pathways, such as mediating inflammatory responses and cell differentiation, and plays a more important role in B cell developmental tumors, rheumatoid arthritis, and X-linked agammaglobulinemia (XLA) [5]. In 2008, Shinohara et al. [6] first discovered that mice lacking Btk showed severe osteopetrosis due to a defect in bone resorption, indicating that Btk is related to bone resorption.

Phospholipase C (PLC) plays an important role in many physiological and pathological processes in organisms, including cell proliferation and differentiation, regulation of the cytoskeleton, apoptosis, and tumor migration, and in recent years, it has been found that PLC also plays a role in the formation of immune cells and osteoclasts. PLC can be divided into 6 categories, each of which has different subtypes, namely, PLC*β*1-4, PLC*γ*1-2, PLC*δ*1-5, PLC*ε*, PLC*ζ*, and PLC*η*1-2. PLC*γ*2 is a subtype of PLC*γ*, and activated PLC*γ*2 generates second messenger IP3 and DAG by hydrolyzing PIP2, thereby transferring extracellular information to downstream effector molecules in cells, and regulating various activities in cells [7–9]. When signal transduction of PLC*γ*2-Ca^2+^-NFATc-1 was downregulated, osteoclast differentiation was inhibited, and bone loss in mice was also inhibited [10].

Studies have also shown that Btk can bind to key residues on PLC*γ* to activate PLC*γ*2; however, little is known regarding the potential role of Btk and PLC*γ*2 in apical periodontitis bone resorption. There were other signaling pathways involved in the alveolar bone resorption in apical periodontitis, such as Wnt/*β*-catenin signaling [11] and Notch signaling pathways [1, 12]. Therefore, this study established a mouse model of apical periodontitis to determine the expression of Btk, PLC*γ*2, and NFATc-1 during the progression of apical periodontitis and analyzed the correlation between the three factors to assess the role of these factors in apical bone resorption. The mechanism of Btk-PLC*γ*2 in apical periodontitis bone destruction was further examined by cell interference to inhibit or enhance the expression of Btk in osteoclasts.

## 2. Materials and Methods

### 2.1. *In Vivo* Experiments

#### 2.1.1. Establishing Chronic Apical Periodontitis in Mice

Twenty male c57BL/6L mice were purchased from the SPF Animal Experimental Center of Dalian Medical University, and the animal experiments were approved by the Ethics Committee of Dalian Medical University. The mice were anesthetized by intraperitoneal injection of 4% chloral hydrate (0.01 mL/g), a 1/4# small ball drill was used to drill the left distal mandibular first molar, and the opened pulp was exposed to the oral environment. Five mice were randomly selected at 1, 2, 3, and 4 weeks after opening the pulp. Following euthanasia, the bilateral mandibles were separated; the left mandible in each mouse was the experimental tissue, and the right mandible was the normal control. The tissues were placed in a carrier containing an optimal cutting temperature (OCT) compound for freezing treatment before being fixed and decalcified. The tissues were washed, and noncontinuous 6 *μ*m thick slices were prepared using a frozen slicer for hematoxylin-eosin (HE) staining and immunohistochemical staining experiments.

#### 2.1.2. Hematoxylin-Eosin Staining

Noncontinuous sections were randomly selected from each group for HE staining to observe apical inflammation. The frozen sections were dried in a constant temperature incubator at 37°C for 60 minutes, washed with phosphate-buffered saline (PBS) solution, stained with hematoxylin for 5 minutes, stained with eosin for 5 minutes, rinsed with a large amount of running water, treated with xylene to transparency, and sealed with neutral gum, and then, images were obtained using a microscope (Olympus (BX-43), Japan).

#### 2.1.3. Immunohistochemical Staining

Five samples were randomly selected from each group, and immunohistochemistry was used to detect the expression of Btk, PLC*γ*2, and NFATc-1 in chronic apical periodontitis in mice. The main steps were as follows: frozen slices were dried at 37°C constant temperature for 60 minutes and washed, fixed with 0.3% hydrogen peroxide solution, washed, and then incubated with primary antibody, Btk (1 : 400; Boster, Wuhan, China); PLC*γ*2 polyclonal antibody (1 : 1000, Elabscience, Wuhan, China); and NFATc-1 (1 : 7500 dilution; Abcam, Cambridge, UK). The slices were placed in a wet box at 4°C overnight and then incubated with a secondary antibody for 60 minutes at room temperature. After washing, DAB was added, counterstained with hematoxylin, xylene was added, and the slices were dried and observed under a microscope. Microscopic observations showed that the cells stained brown were positive cells, each tissue section was randomly observed under a microscope at ×400 magnification, and images of 5 fields of the root apical tissue of the mandibular first molar were obtained. The Btk-NFATc-1 counting method was as follows: Positive staining was performed for optical density analysis using ImagePro Plus 6.0 image processing software, and the average optical density value (OD average) of the positive cells was measured. The PLC*γ*2 counting method was as follows: Two unknown experimenters examined the number of positive cells of PLC*γ*2 per unit area and, after consultation, unified the mean value of 50 fields per sample as the number of positive cells in the sample. Three noncontinuous slices were randomly selected from each tissue.

#### 2.1.4. Enzyme Histochemical Staining

The staining solution was prepared according to the TRAP staining kit instructions (Sigma-Aldrich, St. Louis, MO, USA). Frozen slices were washed with water, placed in a room temperature fixative for 30 seconds, washed with deionized water at 37°C for 30 minutes, stained in a dyeing tank, and protected from light at 37°C. Following incubation for 1 hour in a water bath, the samples were washed with hematoxylin for 2 minutes, rinsed with running water for 5 minutes, sealed with water, observed under a microscope, and photographed with an Olympus (BX-43) optical microscope. From the photograph, the periapical lesion area (*n* = 5) of the maxillary first molar was counted under a high power microscope (×400), and the number of osteoclasts in the lesion area was counted. Three noncontinuous slices were randomly selected from each tissue sample.

### 2.2. *In Vitro* Experiments

#### 2.2.1. Cell Culture

The experimental RAW264.7 cells (ATCC TIB-71) were seeded at a density of 1 × 10^5^/well in a T25 cell culture flask and cultured in high-sugar Dulbecco's modified Eagle's medium (DMEM) containing 10% fetal bovine serum (Gibco, Grand Island, NY, USA), 2 mmol/L glutamine, and antibiotics at 37°C in a humidified atmosphere of 5% CO_2_, and the culture solution was periodically changed.

#### 2.2.2. Osteoclast Induction and Verification

The RAW264.7 cells were harvested in the fourth generation; the cells were digested by trypsin for 3 minutes, and then plated at a density of 30-40%. The cells were induced with 100 ng/L RANKL, and the normal medium was replaced every other day. After 5 days, the cells were harvested for TRAP staining to verify whether osteoclasts had formed. The cells were washed 1-2 times, fixed with 4% paraformaldehyde for 25 minutes, incubated with TRAP staining reagent (Sigma-Aldrich) 2 mL/well, and incubated at 37°C for 1 hour in the dark. Following incubation, the cells were thoroughly washed with double distilled water and counterstained with hematoxylin for 3 minutes, washed with PBS, mounted, and photographed under an inverted microscope. The number of osteoclasts per square centimeter was counted using a light microscope, 3 or more nuclei were stained, and TRAP-positive multinucleated cells were defined as osteoclasts. The level of TRAP mRNA was also determined to further confirm the osteoclast conversion rate.

#### 2.2.3. Btk-Si RNA Transfection

The cells inoculated for 5 days were plated in a six-well plate at a density of 30%-50%. After attachment, the cells were stimulated by the addition of 100 ng/L lipopolysaccharide (LPS) (Sigma-Aldrich) for 24 hours. The culture solution was discarded, and 1 mL/well of DMEM was added. The transfection solution was configured according to the transfection kit instructions. In the experimental group (Si group), 5 *μ*L of Btk-Si RNA, 95 *μ*L Xfect, and 100 *μ*L of transfection reagent were added. The Btk-Si RNA was designed and produced by Shanghai GenePharma Company. In the negative control (NC) group, 5 *μ*L negative control, 95 *μ*L Xfect, and 100 *μ*L transfection reagent were mixed, and in the blank control (MOCK group), only 100 *μ*L of transfection reagent was added. After standing for 15 minutes, the mixture was added to the cells, the normal culture solution was replaced, and the sample was cultured for 4-6 hours at 37°C. After 24 hours, the cells were harvested, total RNA and protein were extracted, and mRNA and protein levels were measured.

#### 2.2.4. Osteoclast Activity Determined by the CCK-8 Assay

Well plates were seeded with a RAW264.7 cell suspension (100 *μ*L/well) and placed in a 5% CO_2_ incubator at 37°C for 5 days. Following addition of the relevant stimulation factors to the different experimental groups, the CCK-8 kit (Dojindo, Shanghai, China) was used to determine the activity of osteoclasts in each group. A mixed solution containing 10 *μ*L of CCK-8 solution and 90 *μ*L of DMEM was added to each well to avoid the generation of bubbles, the plate was placed in an incubator for 1 hour, and the OD values at 450 nm were measured with a microplate reader. The OD values at 0 hours, 24 hours, and 48 hours were measured, respectively.

#### 2.2.5. Real-Time Quantitative Polymerase Chain Reaction (RT-qPCR)

Determination of mRNA expression of TRAP, Btk, PLC*γ*2, and NFATc-1 was as follows: Total RNA from cells in the experimental and control groups were extracted according to the total RNA extraction kit instructions (TaKaRa, Tokyo, Japan) to remove genomic DNA, and the procedure was carried out according to the instructions of the reverse transcription kit and RT-PCR kit (TaKaRa, Tokyo, Japan). The relative change in mRNA was compared using the 2-*ΔΔ* method. The primers for mouse TRAP, Btk, PLC*γ*2, NFATc-1, and internal reference gene GAPDH were synthesized by TaKaRa Co. Ltd., and the primer sequences are shown in Table 1.

#### 2.2.6. Western Blot

Total protein was extracted from cells using lysis buffer containing protease inhibitors (KeyGEN BioTECH, Nanjing, China). Protein concentration was measured using a QuantiPro Bicinchoninic Acid Assay kit (Sigma-Aldrich). Proteins were separated by 15% dodecyl sulfate, sodium salt-polyacrylamide gel electrophoresis (SDS-PAGE) and transferred to polyvinylidene fluoride membranes. The membranes were cut according to protein size to incubate antibodies (rabbit monoclonal antibodies against GAPDH (1 : 7500 dilution; Cell Signaling Technology, Danvers, MA, USA), rabbit polyclonal antibodies against Btk (1 : 400; Boster, Wuhan, China), rabbit polyclonal antibodies against PLC*γ*1 (1 : 7500 dilution; Abcam, Cambridge, UK), and rabbit polyclonal antibodies against NFATc-1 (1 : 7500 dilution; Abcam, Cambridge, UK)) at 4°C overnight. Membranes were incubated with horseradish peroxidase-conjugated anti-rabbit immunoglobulin G antibody (Abcam, MA, USA) for 2 hours after 3 washes. The membranes were added to an enhanced chemiluminescence (ECL) system (Thermo Scientific, Waltham, MA, USA). Protein bands were visualized using a gel imaging system (Bio-Rad, Hercules, CA, USA). Images were analyzed and outputted using Image Lab (Bio-Rad).

### 2.3. Statistical Analysis

The experimental data were analyzed using SPSS 13.0 software. The data were expressed as the mean ± standard deviation. Comparisons between groups were performed by one-way analysis of variance (ANOVA). *P* < 0.05 was considered statistically significant.

## 3. Results

### 3.1. *In Vivo* Experiments

#### 3.1.1. Apical Periodontitis in Mice Was Successfully Established

**Figure 1:**
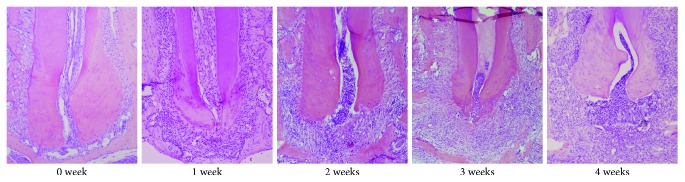
Results of HE staining of periapical tissues in mice at each time point (100x).

The results of HE staining are shown in Figure 1. The periodontal ligament fibers of periapical tissue in the normal control group did not change significantly. Occasionally, mild inflammatory cell infiltration was observed, and no increase in the periodontal ligament was observed. Mild inflammatory cell infiltration occurred in the periapical tissue, and a widening of the periodontal ligament was noted after 1 week. Two weeks after surgery, inflammatory cell infiltration increased with mainly neutrophils, macrophages, and lymphocytes compared with week 1, and alveolar bone absorption was noted. After 3 and 4 weeks, inflammatory cell infiltration was further aggravated, with mostly lymphocytes, accompanied by capillaries and the appearance of fibroblasts, and alveolar bone absorption increased.

#### 3.1.2. Immunohistochemical Staining Results

**Figure 2:**
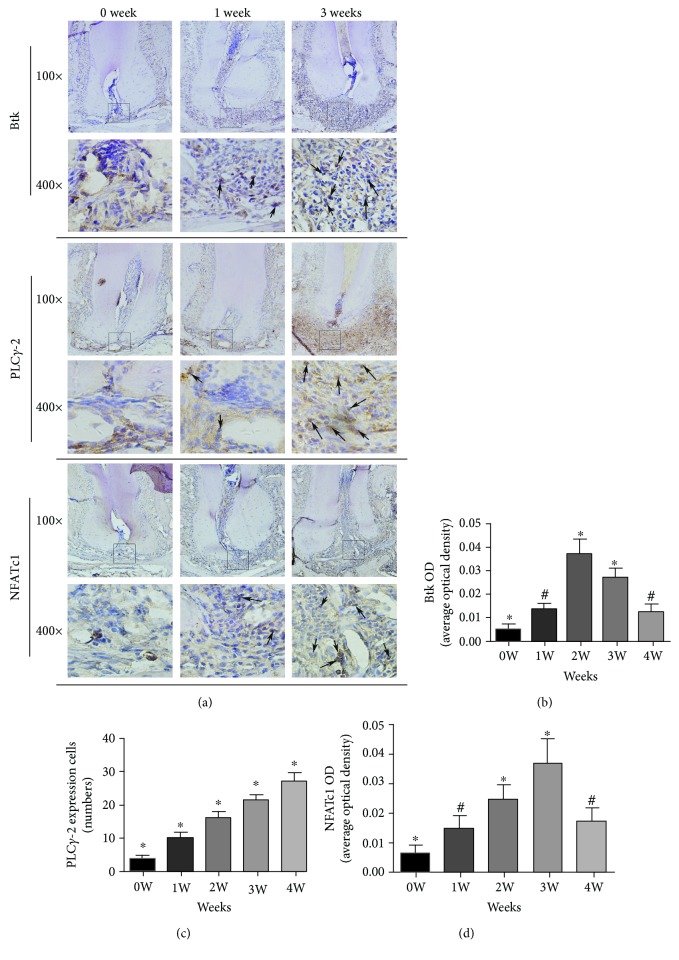
Expression of Btk, PLC*γ*2, and NFATc-1 in periapical tissues of mice at various time points. (a) shows the immunohistochemical staining images of Btk, PLC*γ*2, and NFATc-1 at 0, 1, and 3 weeks, respectively, at ×100 and ×400. (b–d) show the statistical results for the proportion of positive cells at different time points. Black arrows indicate positive cells. ^∗^*P* < 0.05 represents a significant correlation between different groups. ^#^*P* > 0.05 represents no significant correlation between the two groups.

Cells positive for Btk, PLC*γ*2, and NFATc-1 were stained dark brown as shown by immunohistochemistry and were expressed in apical periodontitis at 0 to 4 weeks in experimental mice. The expression of Btk continued to progress with inflammation duration and continued to increase. It reached a peak in the second week (*P* < 0.05) and gradually decreased at 3 weeks (*P* < 0.05) but was still high at 4 weeks; however, there was no significant difference in expression at week 1 and week 4 (*P* > 0.05). The expression of PLC*γ*2 increased with increased inflammatory cell infiltration and alveolar bone resorption (Figure 2(a)). The number of PLC*γ*2-positive cells was statistically different at 0, 1, 2, 3, and 4 weeks (*P* < 0.05), and the highest expression level was noted at 4 weeks. The expression of NFATc-1 increased with increased inflammatory cell infiltration, and the expression level was highest in the third week (*P* < 0.05). There was no significant difference in the expression of NFATc-1 at 4 weeks and 1 week (*P* > 0.05). Positive expression was mainly located in the nucleus of cells such as lymphocytes and plasma cells, with partial expression in the cell membrane and cytoplasm.

#### 3.1.3. Enzyme Histochemical Staining Results

**Figure 3:**
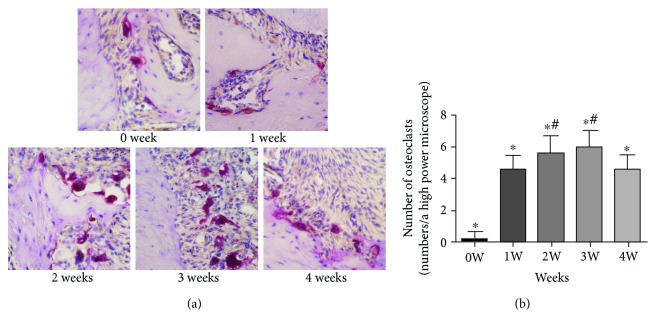
Results of osteoclast staining in mouse periodontal tissues at various time points. (a) The figure shows the results of TRAP staining of periapical tissue in mice at ×400 at each time point. (b) The number of positive osteoclast cells in chronic apical periodontitis in mice. ^∗^*P* < 0.05 represents a significant correlation between the different groups. ^#^*P* > 0.05 represents no significant correlation between the two groups.

The results of TRAP staining (Figure 3) showed that osteoclasts were rarely observed in apical periodontal tissues in the normal control group (0 week). One week after surgery, a small number of polymorphic osteoclasts which were burgundy in color were observed in the cytoplasm; blue nuclei after TRAP staining were observed near the apical area of the alveolar bone, and bone resorption near the trabecular bone was noted. The number of osteoclasts continued to increase in the second week. Three weeks after surgery, the number of osteoclasts peaked, and at 4 weeks after surgery, the number of osteoclasts was observed to decrease, and the number of osteoclasts at weeks 1, 2, 3, and 4 was higher than that in the control group (*P* < 0.05). However, there was no significant difference between the experimental groups at weeks 2 and 3 (*P* > 0.05), but significant differences were observed between the other groups (*P* < 0.05).

### 3.2. *In Vitro* Experiments

#### 3.2.1. Successful Osteoclast Induction

**Figure 4:**
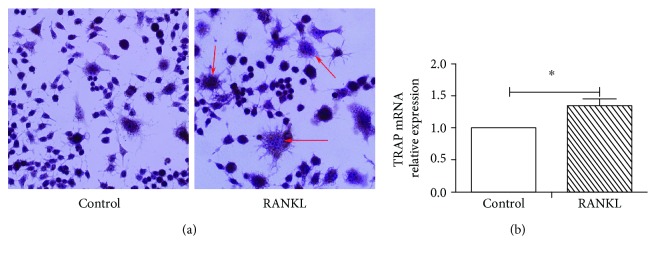
TRAP staining images and the expression of TRAP mRNA: (a) TRAP staining images under an optical microscope; (b) the expression of *TRAP* mRNA in osteoclasts induced by RANKL; ^∗^*P* < 0.05 represents a significant correlation between different groups.

A large number of osteoclast precursor RAW264.7 cells were successfully induced into polymorphic cells with large, round, oval, and dehydrated egg types after 5 days of RANKL induction, and pseudopods were observed around the cells. After TRAP staining, the cells were observed to be burgundy in color under high magnification, with 3 or more nuclei which were not stained and were clearly visible. Numerous dark brown acid phosphatase granules were noted in the cytoplasm (Figure 4(a)). As shown in Figure 4(b), after RAW264.7 cells were induced by RANKL for 5 days, the gene expression level of TRAP was significantly higher than that in the control group (*P* < 0.05).

#### 3.2.2. Effect of Btk on Osteoclast Proliferation in the LPS-Mediated Inflammatory Microenvironment

**Figure 5:**
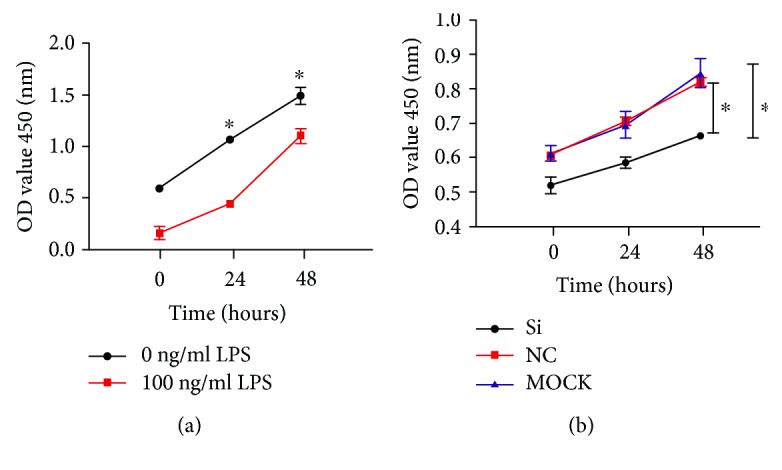
Proliferation of osteoclasts following induction by LPS and Btk-Si RNA transfection: (a) proliferation of osteoclasts after induction by LPS; (b) proliferation of osteoclasts after Btk-Si RNA transfection; ^∗^*P* < 0.05 represents a significant correlation between different groups. MOCK: blank control group; NC: positive control group; Si: experimental group.

Results of the CCK-8 assay are shown in Figure 5(a). 100 ng/L LPS promoted the proliferation of osteoclasts after 24-hour stimulation (*P* < 0.05), and the cell proliferation rate was also significantly higher than that in the control group at 48 hours (*P* < 0.05). Compared with the normal control group and the MOCK group, 100 ng/L LPS was first added to the cells for 24 hours before the addition of Btk-Si RNA, and the results showed that cell proliferation in the Si group was significantly decreased at 24 hours and 48 hours. When Btk was silenced, the proliferation of osteoclasts was inhibited (*P* < 0.05), as shown in Figure 5(b).

#### 3.2.3. Effect of Btk on Osteoclastogenesis in the LPS-Mediated Inflammatory Microenvironment

**Figure 6:**
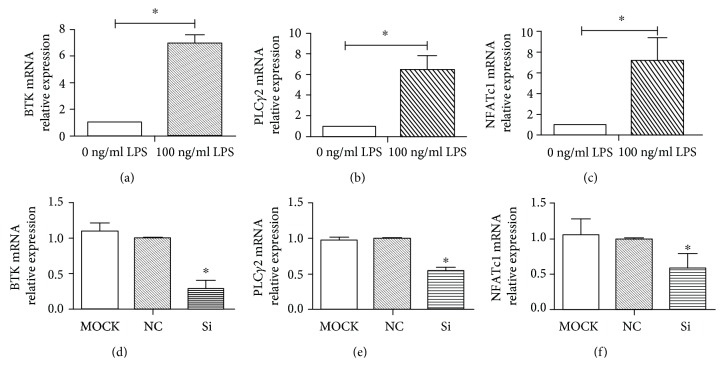
Expression of Btk mRNA, PLC*γ*2 mRNA, and NFATc-1 mRNA: (a–c) the expression of Btk mRNA, PLC*γ*2 mRNA, and NFATc-1 mRNA induced by LPS; (d–f) the expression of Btk mRNA, PLC*γ*2 mRNA, and NFATc-1 mRNA after Btk-Si RNA transfection. ^∗^*P* < 0.05 represents a significant correlation between different groups.

**Figure 7:**
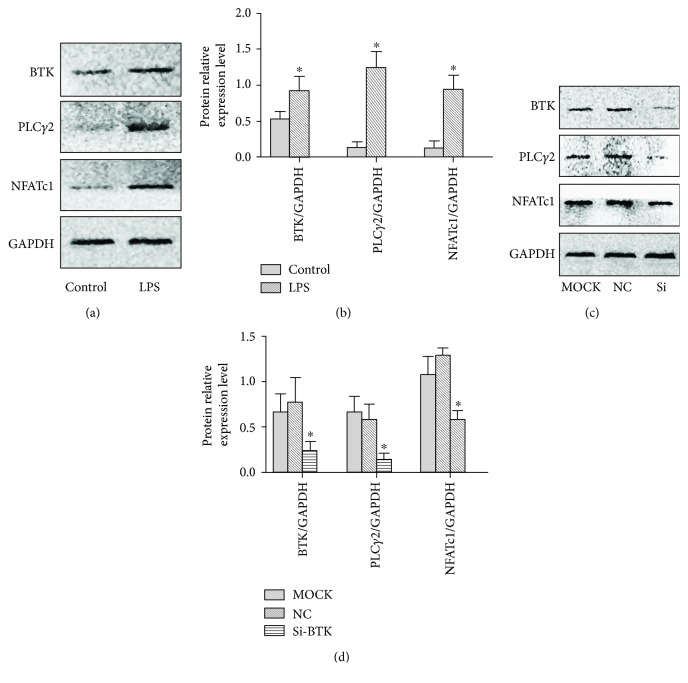
Protein expression of Btk, PLC*γ*2, and NFATc-1. (a, b) The protein expression of Btk, PLC*γ*2, and NFATc-1 in the control and LPS groups and the statistical results. (c, d) The protein expression of Btk, PLC*γ*2, and NFATc-1 in the MOCK, NC, and Si groups and the statistical results. ^∗^*P* < 0.05 represents a significant correlation between different groups.

As shown in Figures 6(a)–6(c), the real-time qPCR results showed that the expression levels of Btk, PLC*γ*2, and NFATc-1 mRNA were upregulated by LPS, and the differences were statistically significant (*P* < 0.05). This indicated that the expression levels of Btk and its downstream factors was significantly increased in osteoclasts in the inflammatory environment, and Btk may be involved in the process of osteoclast inflammation. Twenty-four hours after Btk was silenced (Figures 6(d)–6(f)), the expression of Btk mRNA was significantly decreased (*P* < 0.05), demonstrating that Btk silencing was successful. However, compared with the MOCK group and the normal control group, the expression of PLC*γ*2 and NFATc-1 mRNA was significantly decreased 24 hours after Btk silencing (*P* < 0.05). Even in the LPS inflammatory environment, the differentiation of osteoclasts was significantly inhibited after Btk silencing, indicating that Btk is a key factor in the formation of osteoclasts. The results of Western blotting also confirmed that the expression of Btk protein was significantly decreased after Btk silencing (*P* < 0.05), and the protein expression of PLC*γ*2 and NFATc-1 was also decreased (*P* < 0.05). These results were consistent with the results of gene expression (Figure 7).

## 4. Discussion

Bone tissue homeostasis is regulated by bone-resorbing osteoclasts and bone-forming osteoblasts [13, 14]. Factors affecting the balance of bone formation and bone destruction can lead to bone formation disorders, and massive production of osteoclasts caused by inflammation is the main cause of bone resorption in apical periodontitis. Therefore, determining the relevant factors in osteoclastogenesis is essential in the study of periapical bone destruction.

Osteoclasts are derived from mononuclear or macrophage hematopoietic lines and are highly differentiated multinucleated giant cells. Osteoclasts are the only cell type with bone resorption activity, and the number of osteoclasts can represent bone resorption activity. In this study, a mouse model of apical periodontitis was established using the medullary cavity exposure method. The results of enzyme histochemical staining showed that the number of osteoclasts was the highest at 2 weeks and 3 weeks, indicating that the active period of periapical bone resorption was 2 weeks and continues up to the third week. When inflammation progressed in the fourth week, bone resorption was chronic. Compared with the previous establishment of rat apical periodontitis [15–17], establishment of the mouse apical periodontitis animal model was more complicated but was feasible as many transgenic mouse models are used in research. This study successfully established a mouse model of apical periodontitis to provide a basis for further study of the mechanism of apical periodontitis in transgenic mice.

In the animal experiments, we examined the expression of Btk, PLC*γ*2, and NFATc-1. Btk expression was the highest at 2 weeks, and then gradually decreased with time, indicating that Btk played a role in the acute phase of inflammation progression and the active phase of bone destruction. The expression of PLC*γ*2-positive cells increased during the entire process of apical inflammation in mice, while the number of osteoclasts peaked at 3 weeks and gradually decreased from 3 weeks to 4 weeks. Lymphocyte accumulation is thought to be a protective host response during inflammation, and the purpose of this reaction is to eliminate invading microorganisms and prevent bacteria from directly invading bone tissue around the root tip [18]. Therefore, it can be speculated that PLC*γ*2 plays a role in osteoclast differentiation in the early stage of inflammation. In the later stages of inflammation, its role may be to regulate the differentiation and function of lymphocytes. The master transcription factor NFATc-1 (nuclear factor of activated T-cell cytoplasmic) is essential for the expression of osteoclast-related genes, which are activated by RANKL stimulation in the early phase of osteoclast differentiation [19, 20]. The results of this research showed that the expression of NFATc-1 gradually increased with time and peaked at 3 weeks, and the active phase of bone resorption was also the highest at 3 weeks. These findings demonstrate that NFATc-1 plays a key role in periapical bone resorption. The *in vivo* experiments showed that Btk, PLC*γ*2, and NFATc-1 were expressed at different levels in different stages of apical periodontitis; however, whether these three factors are involved in osteoclastogenesis has not been proven in mouse apical periodontitis.

Btk is mainly involved in the proliferation, differentiation, and apoptosis of B cells but is also closely related to the function of immune cells such as neutrophils, monocytes, and macrophages in the inflammatory response. Numerous studies have proved that Btk can regulate osteoclast maturation by modulating RANKL-mediated NFATc-1 expression. Experiments have shown that blocking Btk signaling can effectively improve experimental arthritis [21]. The orally available Btk inhibitor ibrutinib (PCI-32765) inhibited the formation of osteoclasts by regulating their differentiation, successfully inhibited RANKL-induced bone loss in mice [22], and significantly inhibited mouse multiple myeloma (MM) growth and osteolysis of implanted human bone fragments induced by MM cells [23]. Btk regulation of bone resorption is closely related to PLC*γ*2. Btk binds to key residues on PLC*γ*2 and activates PLC*γ*2, and after activation, PLC*γ*2 catalyzes a series of reactions to release Ca^2+^. An increased Ca^2+^ level can induce NFATc-1 dephosphorylation and NFATc-1 transfer to the nucleus. NFATc-1 regulates osteoclast activity by binding to important genes related to osteoclast differentiation or function, such as tartrate-resistant acid phosphatase (TRAP) and osteoclast-associated receptor (OSCAR) [24–26].

Although the above studies have proved that Btk is a key factor in promoting osteoclastogenesis, especially in murine Btk-deficient osteoclasts, there was a failure in bone formation and activation [27]. But the X-linked agammaglobulinemia (XLA) patients exhibited normal bone turnover parameters, suggesting that humans with XLA, who lack B cells, have an elevated systemic inflammatory status and are especially prone to pneumonia or gastritis [28, 29]. In the presence of inflammation, elevated levels of the proinflammatory factors, TNF-*α*, IL-6, and IL-1*β*, are also a key factor in osteoclast formation [30].

Therefore, we tested the relationship between Btk, PLC*γ*2, and NFATc-1 using *in vitro* experiments and further verified the effect of osteoclast differentiation in the presence of proinflammatory factor LPS. The results confirmed that Btk-Si RNA could still promote the formation of osteoclasts in the presence of LPS, but the rate of formation was lower than that of the control group. Following knockdown of Btk, the mRNA and protein changes in PLC*γ*2 and NFATc-1 were consistent. The results of this study indicate that Btk enhanced the differentiation of osteoclasts in an inflammatory environment, which was mainly achieved by regulating the expression of PLC*γ*2 and NFATc-1. Previous studies have also shown that in addition to the regulation of osteoclast differentiation, Btk can also regulate MAPK, NF-*κ*B, and PKC*α* signaling to suppress osteoblastic differentiation [31].

## 5. Conclusion

In this study, we showed that Btk-PLC*γ*2 is highly expressed during the progression of apical inflammation and bone resorption in mice and is accompanied by high expression of the key transcription factor NFATc-1. Furthermore, *in vivo* experiments showed that Btk is a key factor in osteoclast differentiation, and its ability to promote osteoclast differentiation is greater in the presence of proinflammatory factors, which is mainly achieved by regulating the expression of PLC*γ*2 and NFATc-1. The results of this study provide a new opportunity for the therapeutic study of bone resorption in apical periodontitis, and a Btk inhibitor may be a potential treatment of periapical bone destruction.

## Figures and Tables

**Table 1:** Primer sequences of related genes.

Primer	Sequences
TRAP R	3′-GGGTCACTGCCTACCTGTGT-5′
TRAP F	3′-TCATTTCTTTGGGGCTTATCTC-5′
Btk R	3′-CTGAGAGCAGCAGAGATACTGTCCA-5′
Btk F	3′-AAGGTTCCCGTACCCATTCCA-5′
PLC*γ*2 R	3′-CATGTGACGTTGCTGCTCCA-5
PLC*γ*2 F	3′-GTTGTTCAGGCCATCCGAGAC-5
NFATc-1 R	3′-TCAGCCGTCCCAATGAACAG-5
NFATc-1 F	3′-CAAGTCTCACCACAGGGCTCACTA-5
GAPDH R	3′-TGAAGGGGTCGTTGATGG-5′
GAPDH F	3′-AAATGGTGAAGGTCGGTGTG-5′

## Data Availability

The data used to support the findings of this study are included within the article.
